# Lipoprotein(a) and High-Risk Coronary Plaques: Mechanisms, Characteristics, and Emerging Therapeutic Strategies

**DOI:** 10.31083/RCM44003

**Published:** 2025-10-29

**Authors:** Shuhan Fang, Chancui Deng, Ranzun Zhao

**Affiliations:** ^1^The First Clinical Institute, Zunyi Medical University, 563000 Zunyi, Guizhou, China; ^2^Department of Cardiology, Affiliated Hospital of Zunyi Medical University, 563000 Zunyi, Guizhou, China

**Keywords:** lipoprotein(a), high-risk coronary plaques, mechanisms, atherosclerosis, therapeutic strategies

## Abstract

Lipoprotein(a) (Lp(a)) is an established independent risk factor for atherosclerotic cardiovascular disease, particularly in the development of high-risk coronary plaques (HRPs). Elevated Lp(a) contributes to lipid accumulation, vascular inflammation, and plaque instability, primarily through oxidized phospholipids that promote monocyte adhesion and foam cell formation. Genetic studies have identified variants in the* LPA* gene as major determinants of Lp(a) levels, with higher concentrations consistently associated with adverse cardiovascular outcomes. Intravascular imaging techniques, such as optical coherence tomography and intravascular ultrasound, along with coronary computed tomography angiography (CCTA), have confirmed strong correlations between elevated Lp(a) and increased plaque burden, lipid-rich necrotic cores, and thin fibrous caps. In addition to coronary involvement, Lp(a) is implicated in systemic atherosclerosis, contributing to peripheral artery disease, cerebrovascular disease, and calcific aortic stenosis. Although conventional lipid-lowering therapies exert minimal effects on Lp(a), novel treatments such as proprotein convertase subtilisin/kexin type 9 inhibitors and RNA-targeted agents offer promising approaches to mitigating Lp(a)-mediated risk. This review summarizes current insights into the pathophysiological role of Lp(a) in HRP formation and progression, integrating evidence from genetic, mechanistic, and imaging studies, while highlighting emerging therapeutic strategies. Nonetheless, continued research is essential to enhance our understanding of Lp(a)-driven plaque vulnerability and to inform precision-targeted cardiovascular prevention.

## 1. Introduction

Coronary high-risk plaques (HRPs) are considered the most critical potential lesions associated with acute coronary syndrome (ACS). Studies have indicated that patients with HRPs are 2 to 4 times more likely to experience future cardiovascular events than those without such plaque [1]. Both domestic and international guidelines consistently emphasize that achieving target levels of low-density lipoprotein (LDL-C) is the primary therapeutic goal for these HRPs [2]. However, even when LDL-C treatment reaches its target, residual cardiovascular risk remains significant, involving factors such as residual cholesterol risk, elevated triglycerides, impaired high-density lipoprotein (HDL-C) function, oxidative stress, inflammation, and metabolic factors like diabetes, insulin resistance, and obesity [2].

In the past few years, lipoprotein(a) (Lp(a)), a large glycoprotein attached to a low-density lipoprotein-like particle, has attracted increasing attention from researchers [3]. Multiple studies have indicated that elevated Lp(a) levels are an independent and heritable causal risk factor for atherosclerotic cardiovascular disease (ASCVD) [4]. Lp(a) plays a central role in lipid metabolism and is strongly associated with the development of atherosclerosis (AS) and HRPs. Furthermore, elevated Lp(a) levels can predict early-onset atherosclerotic vascular disease and influence coronary heart disease (CHD) risk in hypercholesterolemic patients [4]. Many authoritative consensus statements recommend incorporating Lp(a) into global cardiovascular risk assessment [5]. Therefore, grasping the role of Lp(a) in the development and progression of HRPs holds significant clinical value for reducing major adverse cardiovascular events (MACEs). This article provides a systematic review of the current understanding of Lp(a), its role, and related mechanisms in HRP formation, as well as relevant basic and clinical research, therapeutic strategies, and challenges.

## 2. The Physicochemical Features of Lipoprotein (a)

Lp(a) was first identified and named in 1963 by Norwegian geneticist Kare Berg and Mohr J [6]. It is a liver-derived lipoprotein similar in structure to LDL; however, unlike LDL, it covalently binds to a unique apolipoprotein (a) (apo(a)) subunit via disulfide bonds [7]. Its lipid core mainly consists of cholesterol esters and triglycerides, while its outer layer includes phospholipids, free cholesterol, and apoB-100. Research indicates that Lp(a) plasma levels are primarily regulated by genetic factors, with significant variation among individuals (ranging from 1 to 200 mg/dL in the general population). Additionally, Lp(a) concentrations differ across racial/ethnic groups, and the relationship between Lp(a) levels and cardiovascular disease risk may vary by ethnicity [8]. But the UK Biobank paper by Amit Khera demonstrates that although levels of Lp(a) differ by ancestry, the relationship to ASCVD is the same when the data are plotted as a percentage of the population [9].

The pathophysiological roles of Lp(a) are mainly driven by its apo(a) subunit. The *LPA* gene, located on chromosome 6 at *6q2.6-2.7*, encodes apo(a), which shares significant homology with plasminogen [10]. Apo(a) contains multiple kringle domains, including genetically variable kringle IV type 2 (*KIV2*) repeats, which significantly affect Lp(a) levels [11]. The number of *KIV2* repeats correlates with higher Lp(a) levels and greater cardiovascular risk. Variations in apo(a) size, driven by *KIV2* repeat variation, contribute to its genetic diversity [12]. Additionally, oxidized phospholipids (OxPL) bind with *KIV10*, influencing apo(a) structure and its functional consequences on Lp(a) metabolism [13]. Changes in the number of *KIV2* structures inversely correlate with liver production rates, and larger isomers exhibit negative correlations with plasma Lp(a) concentrations, this is likely due to extended intracellular processing that results in enhanced degradation of larger isomers [14].

The quantity of kringle domains in an individual is determined by the genetic information inherited from each parent. Single-nucleotide polymorphisms (SNPs), which represent single-nucleotide changes at specific locations within a gene, contribute to genetic specificity and polymorphism [15]. Two large mendelian randomization studies have demonstrated that polymorphic variants of the *LPA* gene (e.g., *rs3798220* and *rs10455872*) are directly strongly linked to increased Lp(a) levels and a higher risk of CHD, providing genetic support for the causal relationship between Lp(a) and CHD pathogenesis, suggesting that SNPs in the *LPA* gene play a crucial role in the formation of HRPs [11, 16].

## 3. The Pathological Mechanism of Lipoprotein(a) Involved in the Occurrence and Progression of Atherosclerosis 

Lp(a) is a key contributor to the onset and progression of AS, functioning similarly to LDL-C as an initiating factor [4]. Lp(a) migrates to the arterial wall, accumulating beneath the endothelium and forming lipid streaks that initiate atherosclerosis [17]. Notably, due to its high lipophilicity, Lp(a) accumulates more extensively than LDL-C. Within the arterial wall, Lp(a) predominantly concentrates in the extracellular intima and subintima, where it anchors via interactions between its lipoprotein structure and lysine binding sites of apolipoprotein [13]. Lp(a) enhances inflammation through OxPLs and promotes vascular lipid deposition [13]. The latter interaction explains the differing affinities of Lp(a) and LDL for the arterial wall, potentially contributing to AS progression [18]. Additionally, α-defensins derived from neutrophils serve as potential ligands for Lp(a), forming stable complexes within atherosclerotic plaques that remain extracellular, representing a mechanism for Lp(a) aggregation. Despite this, Lp(a) can also be internalized into cells, promoting foam cell formation in macrophages. Through either direct entry or heparin-mediated mechanisms, Lp(a) enhances lipid-driven processes, thereby exacerbating AS. Increased cholesterol content in macrophages promotes the calcium-dependent internalization and degradation of Lp(a) and apo(a), independent of LDL clearance rates, LDL receptor-related proteins, plasminogen receptors, or cell membrane glycoproteins [19]. This process involves the coordinated actions of the extracellular matrix, enzymes, macrophages, and vascular smooth muscle cells (VSMCs). Sphingomyelinase released by macrophages and lipoprotein lipase synergistically promote the adhesion of LDL and Lp(a) to aortic SMCs and the extracellular matrix. Furthermore, Lp(a) may be preferentially absorbed by macrophage scavenger receptors [20].

Lp(a) regulates inflammatory cell aggregation within the vascular wall, facilitating AS progression. It upregulates the expression of adhesion molecules, such as vascular cell adhesion molecule-1 (VCAM-1) in coronary artery endothelial cells (ECs), as well as intercellular adhesion molecule-1 (ICAM-1) in human umbilical vein ECs. This effect is partly attributed to Lp(a)’s inhibitory action on transforming growth factor-β (TGF-β) [21]. Additionally, Lp(a) and β2-integrin macrophage-1 antigen (Mac-1) jointly promote monocyte adhesion and infiltration [22]. Lp(a) activates monocytes via Toll-like receptor (TLRs) and nuclear factor kappa B (*NFκB*) signaling, driving tissue factor (TF) expression and activity, amplifying immune thrombosis risks through mechanisms involving TLRs, *NFκB*, and monocyte TF, and promoting HRPs formation [23]. Moreover, Lp(a) exhibits chemotactic properties, with plasminogen and inactive plasmin inhibiting its effects on monocyte migration. This suggests that the lysine binding sites of apo(a) play a critical role. Lp(a) accelerates chemotaxis by promoting endothelial secretion of monocyte chemoattractant protein and interleukin-8 (IL-8), increasing neutrophil infiltration [24]. Studies indicate that IONIS-APO(a)Rx treatment effectively reduces Lp(a) levels; however, exogenous Lp(a) addition does not affect fibrinolytic function in low-Lp(a) plasma, whereas recombinant apo(a) exhibits anti-fibrinolytic activity, suggesting that the atherogenic effects of Lp(a) are mediated by apo(a), which inhibits fibrinolysis, increases plaque vulnerability, and promotes thrombotic events [25]. Lp(a) also enhances the expression of interleukin-1β (IL-1β) and tumor necrosis factor-α (TNF-α) in macrophages, exacerbating inflammation in the arterial wall. By binding to plasminogen, Lp(a) blocks its conversion to plasmin, reducing blood fibrinolytic activity and suppressing plasmin-mediated TGF-β activation, which normally inhibits smooth muscle cell growth. Lp(a) prevents plasminogen binding to endothelial cells, platelets, fibrin, and monocytes [26].

Lp(a), as the primary carrier of OxPLs, plays a detrimental role in AS, vascular inflammation (VI), thrombosis, and endothelial dysfunction, contributing to cardiovascular events and the development of HRPs [27]. Although OxPLs are mainly generated through LDL oxidation, they are closely associated with Lp(a) *in vivo*. Low levels of Lp(a) help remove OxPLs from plasma and promote their degradation via *Lp-PLA2*, whereas high Lp(a) levels lead to excessive OxPL accumulation in the arterial wall, promoting macrophage apoptosis and plaque necrosis [28]. Lp(a) activates inflammatory pathways and lipid deposition through OxPLs, establishing a “pro-inflammatory - pro-oxidative - pro-thrombotic” vicious cycle. Studies show that OxPLs carried by Lp(a) activate endothelial cells, induce transendothelial migration of monocytes, and promote atherosclerosis [29]. Transcriptomic analysis reveals that Lp(a) drives endothelial pro-adhesive states through *PFKFB3*-dependent glycolysis [20]. Elevated Lp(a) levels correlate with the distribution of pro-atherogenic monocyte subsets in patients with stable AS, and the involvement of *OxPL/apoB* suggests that this may represent a potential therapeutic target for cardiovascular disease [27].

**Fig. 1.**
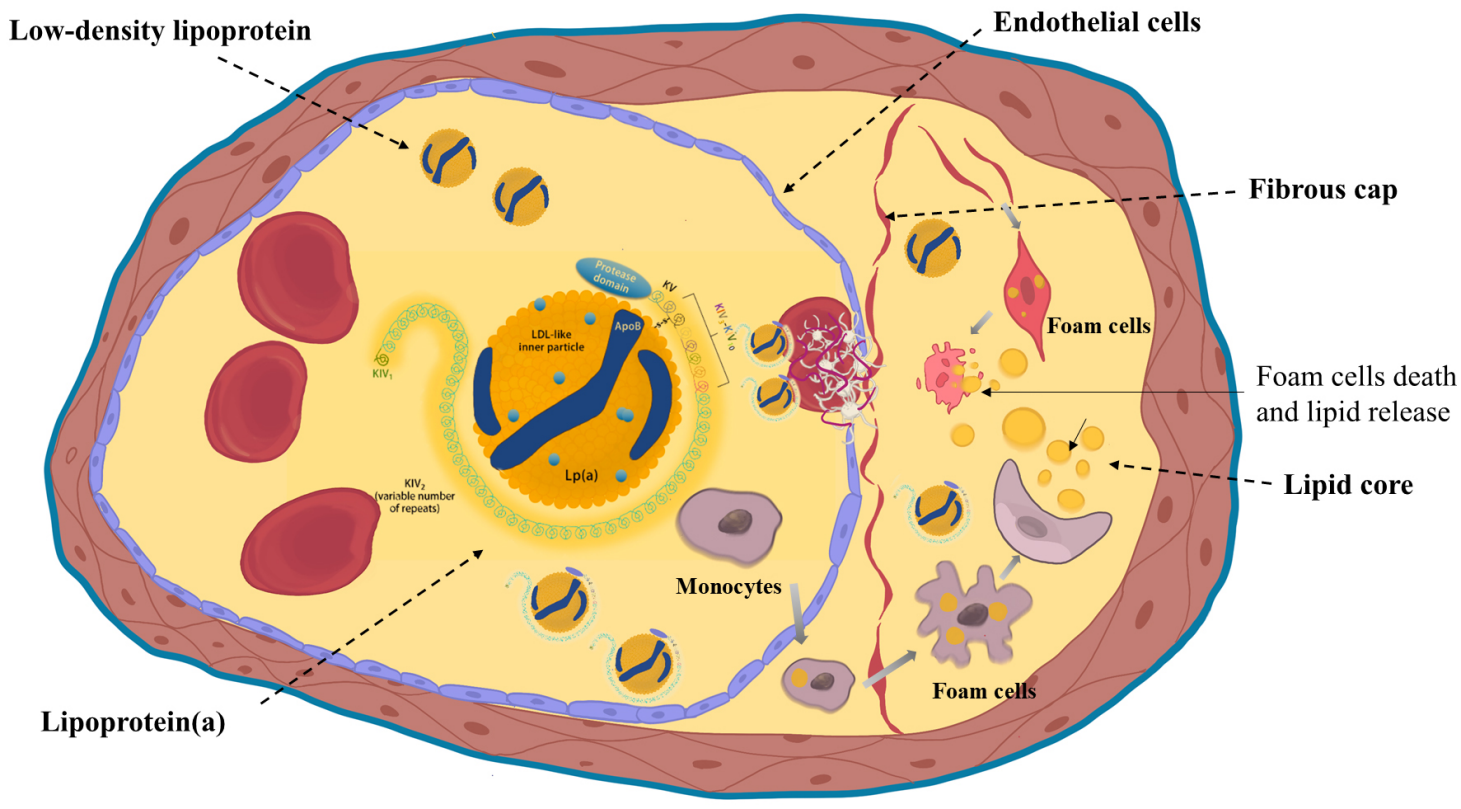
**Structure of lipoprotein(a) and its atherogenic mechanisms**. Lipoprotein(a) (Lp(a)) consists of an LDL-like particle and apo(a) rich in kringle domains. It promotes endothelial dysfunction, macrophage-mediated inflammation, and foam cell formation, contributing to atherogenesis. Created with Procreate. KIV, kringle IV; LDL, Low Density Lipoprotein; KV, kringle V; ApoB, apolipoprotein B.

Lp(a) affects plaque stability through an increase in the expression of micro-PAR and ICAM-1, which affects plaque stability and leads to monocyte binding, resulting in an enlarged lipid core and a thinner fibrous cap in the plaque. Thus, Lp(a) plays a key role in the initiation of AS and plaque instability [21]. Collectively, these proatherogenic and proinflammatory actions of Lp(a) accelerate plaque progression and destabilization, as illustrated in Fig. 1. These molecular insights form the mechanistic foundation for understanding imaging manifestations of Lp(a)-related plaque vulnerability, which will be discussed in the following section.

## 4. New Understandings of the Function of Lipoprotein(a) in Coronary High-Risk Plaques 

Post-mortem studies have shown that plaque rupture is the main pathological change in acute MI, though the mechanism of transition from chronic coronary syndrome to ACS remains unclear. The concept of “vulnerable plaque”, introduced in 1994 by Muller *et al*. [30], refers to plaques at high risk of rupture despite not causing significant stenosis. Key features include large lipid cores, thin fibrous caps, and inflammation. Rupture of such plaques can induce thrombosis, leading to MACEs, including myocardial infarction (MI) or stroke. In 2003, Naghavi *et al*. [31] defined vulnerable plaques as “thrombogenic plaques with a high probability of progression to culprit plaques”. As clinical understanding advances, the term “high-risk plaque” has replaced “vulnerable plaque”, now encompassing not only plaque fragility but also systemic risk factors: vulnerable blood (hypercoagulable state due to imbalance between coagulation and fibrinolysis) and vulnerable myocardium (electrophysiological instability) [32]. This conceptual evolution marks a paradigm shift in clinical diagnosis and treatment from “local plaque intervention” to “systemic risk assessment”.

The advancement of plaque risk assessment has been driven by imaging innovations. Coronary angiography, once the primary risk indicator, showed that 60% of acute coronary events arise from the rupture of non-severe stenotic plaques, revealing the limitations of purely anatomical assessments [33]. Intravascular ultrasound (IVUS) and optical coherence tomography (OCT) have revealed the *in vivo* microscopic characteristics of HRPs, including lipid core >40%, fibrous cap thickness <65 µm, macrophage infiltration, and positive remodeling (>5% increase in external elastic membrane area) [33]. Coronary computed tomography angiography (CCTA) has been used as a non-invasive screening method to identify high-risk plaques using four markers: ① Positive remodeling: the remodeling index (RI) is derived by dividing the cross-sectional area of the narrowest portion of the vessel by the average cross-sectional area of the proximal and distal reference segments. Positive remodeling (RI ≥1.05) and negative remodeling (RI <0.95). ② Low CT attenuation: Non-calcified plaques with a mean CT value <30 HU indicate low attenuation. ③ Napkin-ring sign: A central lesion with low CT attenuation surrounded by a ring of slightly higher attenuation. ④ Spotty calcification: These are small calcified deposits with a diameter less than 3 mm in the dual source CT (DSCT) field of view. Their length does not exceed 1.5 times the lumen diameter, and their width remains within two-thirds of the lumen diameter. The presence of any one of these characteristics can define an HRP, with a positive predictive value exceeding 85%. Emerging technologies like near-infrared spectroscopy (NIRS) quantify lipid core burden index (LCBI), enabling precise plaque component analysis. Multimodal imaging fusion is advancing risk assessment from static to dynamic evaluation [34].

The instability of coronary HRPs is primarily influenced by local and systemic inflammation, blood flow shear stress, and matrix metalloproteinase activity [35]. HRPs include thin-cap fibroatheromas (TCFA) and calcified nodules, both of which are linked to plaque rupture, erosion, and coronary events. Plaque progression is characterized by VSMC apoptosis, matrix degradation, angiogenesis, arterial remodeling, fibrous cap rupture and thrombosis, as well as necrosis and calcification. Importantly, plaque vulnerability is a dynamic rather than a static phenomenon. With optimal medical therapy, as many as 75% of HRPs may stabilize over time, losing their high-risk characteristics. Conversely, in a certain proportion of patients, stable plaques may evolve into morphologically more fragile plaques. Studies have shown that inflammation is a crucial factor in both plaque development and rupture, especially through the infiltration of macrophages and T cells [35].

Coronary plaques continuously evolve through processes such as plaque hemorrhage, erosion, or rupture, which is a dynamic process. However, almost all technical methods for early identification of vulnerable plaques perform vascular imaging at a single time point. Even if the imaging modality can identify HRPs, it does not imply that treating a single plaque, such as placing a stent within such a plaque, can reduce subsequent morbidity and mortality. The ISCHEMIA (N Engl J Med 2020) study included 5179 patients with stable CHD. The findings suggested that traditional interventional treatment was not more effective in reducing MACEs when compared to drug therapy alone, implying that local stent implantation does not impact the systemic atherosclerotic process [36]. Steven Nissen pointed out that the concept of waiting for the rupture of a specific vulnerable plaque is overly simplistic. In fact, treatments proven to reduce coronary events are systemic, such as lipid-lowering therapy, antiplatelet therapy, and anti-inflammatory therapy. This suggests that the treatment concept focused on a single “vulnerable plaque” is no longer suitable. The essence of treatment lies in influencing the various factors involved in plaque development, and the comprehensive implementation of integrated intervention measures is of greater significance.

Lp(a) plays a key role in the development of high-risk coronary plaques by promoting atherosclerosis, thrombosis, inflammation, and plaque instability. A meta-analysis of 18 studies involving 23,105 asymptomatic patients (average age 55.9 years, 46.4% female) showed that elevated Lp(a) levels were significantly associated with the risk of coronary artery calcification (CAC). Specifically, for every 1 mg/dL increase in Lp(a), the risk of CAC >0 increased by 1% (Odds Ratio (OR) = 1.01, 95% Confidence Interval (CI) 1.01–1.01). This study was the first to quantitatively confirm in an asymptomatic population that Lp(a) is dose-dependently and positively correlated with the degree and dynamic progression of coronary artery calcification. Despite high heterogeneity, the results provided imaging evidence for the atherosclerotic effects of Lp(a), supporting its inclusion in the risk assessment system for primary prevention of cardiovascular disease (CVD) and providing an evidence-based basis for formulating early intervention strategies for high-risk populations [37].

In addition, IVUS imaging results have shown that even after LDL-C levels have been reduced to below 70 mg/dL or extremely low to <40 mg/dL, only a minor reduction in atherosclerotic plaque burden was observed. This discrepancy between clinical benefit and the degree of plaque burden reduction as measured by IVUS suggests that intensive lipid-lowering therapy (statin therapy combined with proprotein convertase subtilisin/kexin type 9 (PCSK9) inhibitors) may exert beneficial effects on plaque composition beyond simple reductions in plaque volume. The PACMAN-AMI study demonstrated that one-third of acute myocardial infarction (AMI) patients receiving intensive lipid-lowering therapy achieved triple reversal (i.e., reduction in plaque volume, decrease in lipid core burden index, and increase in fibrous cap thickness) [38]. PCSK9 monoclonal antibody treatment was identified as the strongest independent predictor of triple reversal. PCSK9 monoclonal antibodies represent one of the emerging therapies for reducing Lp(a), with significant effects, and have entered phase III clinical trials. Moreover, patients achieving “triple reversal” exhibited a lower risk of MACEs within one year, primarily manifested as a reduction in ischemia-driven revascularization. Another study suggests that elevated Lp(a) may serve as an independent predictor of post-AMI MACE risks, particularly for long-term assessment in women with diabetes or hypertension [39]. These findings underscore the indispensable role of Lp(a) in coronary HRPs and highlight the significance of intensive lipid-lowering therapy in reducing MACEs [40].

High levels of Lp(a) (>50 mg/dL) are a significant risk factor for CVD, strongly linked to cardiovascular calcification, and may promote microcalcification by releasing calcifying extracellular vesicles (EVs). Experiments have shown that Lp(a) can significantly enhance the calcification activity of human primary SMCs and VICs, potentially mediated through an oxidized phospholipid-induced pro-inflammatory mechanism and inhibited by the specific neutralizing antibody *E06*. Using a self-developed single-vesicle microarray detection technology, it was first confirmed at the single-vesicle membrane level that Lp(a) can alter the composition of EV subpopulations and selectively increase the release of *CD29+/tetraspanin-microvesicles*. In a cell-free 3D collagen hydrogel model simulating atherosclerotic plaques and calcified aortic valve extracellular matrix, EVs induced by Lp(a) exhibited significant ectopic calcification ability. Mechanistically, this reveals a novel mechanism through which Lp(a) mediates cardiovascular calcification through regulating EV subpopulations [41], further supporting the direct association between elevated Lp(a) and plaque instability. Collectively, these clinical research findings indicate that elevated Lp(a) levels are closely related to the formation and instability of coronary HRPs.

Research on high-position coronary artery plaques has advanced significantly. The integration of basic and clinical studies has facilitated early identification and intervention strategies for these plaques. Furthermore, Lp(a), as an important biomarker, warrants further exploration regarding its role in the formation of non-coronary HRPs.

## 5. Imaging Modalities-Based Insights into the Association Between Lipoprotein(a) and Coronary Plaque Characteristics

Imaging Modalities-Based Insights into the Association Between Lipoprotein(a) and Coronary Plaque Characteristics. Integrating findings from OCT, IVUS, and CCTA studies provides structural validation of Lp(a)-driven plaque vulnerability (Table 1, Ref. [42, 43, 44, 45, 46, 47, 48, 49, 50, 51, 52]).

**Table 1.** **Clinical studies associated with Lp(a)**.

Study names	Diagnosis of enrollment	Column count and grouping	Research methods	Content of follow-up	Results
Yuan X, *et al*. [42]	Patients with 125 DES-ISR lesions	High Lp(a): (≥30 mg/dL): 47 patients	No intervention	ISNA and TCFA incidence; ROC AUC	Lp(a) identified as an independent predictor of ISNA (OR = 1.054, *p * < 0.001)
		Low Lp(a): (<30 mg/dL): 72 patients	Retrospective, single-center observational study		
Erlinge D, *et al*. [43]	MI within 4 weeks, all culprit lesions successfully treated with PCI	N = 865	PCI or NIRS + IVUS	Clinical tracking; no formal imaging or MACE	Lp(a) was uniquely associated with focal vulnerable plaques, while TC, LDL-C, non-HDL-C, and triglycerides showed no significant associations. These findings suggest Lp(a) contributes to plaque instability rather than plaque burden.
		Low Lp(a): <75 nmol/L	follow-up at 1, 6, 12 months, and annually (median 3.7 years)		
		Intermediate Lp(a): 75–125 nmol/L			
		High Lp(a): >125 nmol/L			
Fathieh S, *et al*. [44]	Adults from the BioHEART undergoing CCTA for suspected CAD	N = 1718	Cross-sectional study; no longitudinal follow-up	CACS; Gensini Score; Plaque morphology (calcified, non-calcified, mixed)	Elevated Lp(a) (>22 nmol/L) is strongly linked to greater CAD severity and mixed plaques, enhancing risk prediction—especially in low/moderate-risk individuals.
		Low Lp(a): ≤22 nmol/L			
		High Lp(a): >22 nmol/L			
Niccoli G, *et al*. [45]	Consecutive patients with ACS and obstructive CAD	CCTA (n = 500)	No intervention	Coronary lesion extent (Sullivan score, Bogaty score), plaque characteristics (lipid radian, TCFA)	Prevalence of lipid plaque in the High Lp(a) ↑ (67% vs 27%, *p* = 0.02)
		OCT (n = 51)	Cross-sectional study		Lipid radian ↑ (135 ± 114 vs 59 ± 111, 0.03)
		High Lp(a): ≥30 mg/dL	Baseline assessment		TCFA proportion ↑ (38% vs 10%, *p* = 0.04)
Yu MM, *et al*. [46]	Patients with stable chest pain (CCTA examination)	Derivation cohort: prospectively enrolled (n = 5607) Validation cohort: contemporaneous retrospective enrolled (n = 1122)	No intervention	Primary endpoint: fatal or nonfatal MI	High Lp(a) levels were independently linked to MI risk (HR 1.91, *p * < 0.001). A significant interaction existed between Lp(a) and LAP. In LAP-positive individuals, elevated Lp(a) markedly increased MI risk (HR 3.03, *p * < 0.001), with LAP mediating 73.3% of this effect. Results were consistent in the validation cohort.
		High Lp(a): Lp(a) ≥50 mg/dL	Observational analysis		
			Median follow-up 8.2 years (Q1–Q3: 7.2–9.3 years)		
Mszar R, *et al*. [47]	Eligibility criteria: Asym*p*tomatic adults (40–65 years old)	N = 1795	No intervention	Coronary CAC >0	Elevated levels of Lp(a) were independently linked to the presence of any coronary plaque: (OR = 1.40, 95% CI: 1.05–1.86) ≥2 high-risk characteristics (OR = 3.94, 95% CI: 1.82–8.52) in the CAC = 0 subgroup, plaque prevalence ↑ (24.2% vs 14.2%, *p * < 0.001).
	Baseline CCTA was performed. Not receiving any therapy for lowering lipids	High Lp(a): Lp(a) ≥125 nmol/L	Cross-sectional analysis	The maximum stenosis was ≥50%	
		Low Lp(a): Lp(a) <125 nmol/L	A single baseline assessment	≥2 HRP features (positive remodeling, punctate calcification, hypoattenuation plaque, napkin ring sign)	
O’Toole T, *et al*. [48]	Stable chest pain and no known coronary artery disease (data on Lp(a) measurements obtained on CTA).	N = 1815	No intervention	Coronary stenosis (≥50%, ≥70%) and HRPs	Elevated Lp(a) showed an independent association with obstructive CAD (OR = 1.40, *p * < 0.05), irrespective of LDL-C levels. However, when accounting for obstructive CAD, high Lp(a) was not linked to HRP.
		High Lp(a): Lp(a) ≥50 mg/dL.	Secondary analysis		
		Low Lp(a): Lp(a) <50 mg/dL.	Short-term follow-up		
		LDL-C stratification: ≥100 mg/100 mL vs. <100 mg/100 mL			
Kaiser Y, *et al*. [49]	Patients with advanced stable CAD.	N = 191	No intervention	Assessment of plaque type (total, low-attenuation, calcified, non-calcified)	The plaque with low attenuation (necrotic core) in the High Lp(a) showed significant annual progression ↑ (26.2 ± 88.4 mm^3^ vs –0.7 ± 50.1 mm^3^, *p* = 0.020)
		High Lp(a): Lp(a) ≥70 mg/dL median 100 mg/dL)	Longitudinal observation		For every 50 mg/dL of Lp(a), necrotic core progression ↑ is 10.5% (95% CI: 0.7%–20.3%).
		Low Lp(a): Lp(a) <70 mg/dL (median 10 mg/dL)	Baseline and 12-month follow-up		
Berman AN, *et al*. [50]	Individuals aged ≥18 with at least one Lp(a) result.	N = 16,419	No intervention	MACEs are defined as non-fatal MI, nonfatal ischemic stroke, coronary revascularization, and cardiovascular mortality.	Lp(a) independently predicts MACE in both primary and secondary prevention, with distinct risk thresholds for each group.
		Based on Lp(a) percentile groups	Retrospective cohort study Median follow-up: 11.9 years (IQR: 6.2–14.4 years)		
		Baseline ASCVD with a history of ASCVD or not			
Nurmohamed NS, *et al.* [51]	Patients with suspected CHD underwent baseline and repeat CCTA after 10 years.	N = 267	Repeated CCTA imaging follow-up	Plaque volume percentage change, LDNP, PCAT	Percentage of plaque volume in the high Lp(a) group ↑ (6.9% vs 3.0%, *p* = 0.01) per doubling of Lp(a), plaque volume ↑ 0.32% (95% CI: 0.04–0.60). PCAT inflammation index continued ↑.
		High Lp(a): ≥125 nmol/L (median threshold)			
Wang X, *et al*. [52]	Patients undergoing CCTA	N = 1618	No intervention	Unstable plaque: based on CCTA image features	The hLp(a)/NLR+ (both elevated Lp(a) and NLR) group had the highest risk of ASCVD (OR = 2.39, *p * < 0.001).
		High Lp(a): plasma Lp(a) >75 nmol/L	Cross-sectional analysis of a single assessment		Incidence of unstable plaque ↑ (OR = 1.67,*p* = 0.035).
		High NLR: NLR >1.686			

DES-ISR, drug-eluting stent in-stent restenosis; ISNA, in-stent neoatherosclerosis; TCFA, thin-cap fibroatheromas; ROC, Receiver Operating Characteristic; AUC, Area Under the Curve; MI, myocardial infarction; PCI, percutaneous coronary intervention; NIRS, near-infrared spectroscopy; IVUS, intravascular ultrasound; MACE, major adverse cardiovascular event; TC, Serum total cholesterol; LDL-C, low-density lipoprotein; HDL-C, high-density lipoprotein; CCTA, coronary computed tomography angiography; CAD, Coronary artery disease; CACS, Coronary Artery Calcium Score; ACS, acute coronary syndrome; OCT, Optical Coherence Tomography; LAP, low-attenuation plaque; CAC, coronary artery calcification; HRPs, high-risk coronary plaques; ASCVD, atherosclerotic cardiovascular disease; CHD, coronary heart disease; LDNP, Low-density non-calcified plaque; PCAT, Pericardial adipose tissue; NLR, Neutrophil to Lymphocyte Ratio.

### 5.1 Lp(a) and Plaque Vulnerability

OCT, with its high resolution (10–20 µm), enables precise visualization of hallmark features of vulnerable plaques, such as thin fibrous caps, lipid cores, macrophage infiltration, and neovascularization. In a study involving 125 drug-eluting stent in-stent restenosis (DES-ISR) lesions, Yuan *et al*. [42] found that patients with elevated Lp(a) levels (≥30 mg/dL) had significantly higher rates of in-stent neoatherosclerosis (ISNA; 94.0% vs. 52.0%, *p* < 0.001) and thin-cap fibroatheroma (TCFA; 42.0% vs. 5.3%, *p* < 0.001). These patients also exhibited increased lipid burden, wider lipid arcs, enhanced neovascularization, and more inflammatory cell infiltration, indicating a potential role of Lp(a) in promoting focal inflammation and lipid accumulation, thereby exacerbating plaque instability and contributing to late stent failure.

### 5.2 Lp(a) and Focal High-Risk Plaques

In a subanalysis of the PROSPECT II study, Erlinge *et al*. [43] employed near-infrared spectroscopy and intravascular ultrasound (NIRS-IVUS) to evaluate coronary plaque characteristics in 865 patients post-myocardial infarction. Although Lp(a) was not associated with overall plaque burden, higher Lp(a) levels were significantly linked to focal high-risk features, such as max LCBI 4 mm ≥324.7 and plaque burden ≥70% (*p* < 0.01). In contrast, LDL-C was associated with diffuse plaque burden but not with these high-risk features. These associations, independent of traditional lipid parameters, underscore Lp(a)’s distinct contribution to plaque vulnerability, possibly through mechanisms involving oxidative phospholipid deposition and activation of vascular macrophages and smooth muscle cells.

### 5.3 Lp(a) and Mixed or Low-Attenuation Plaques

Coronary computed tomography angiography (CCTA) provides noninvasive plaque characterization, including identification of calcified, non-calcified, and mixed plaques. Several studies have highlighted strong associations between elevated Lp(a) and morphologic features of vulnerable plaques. In the BioHEART study, Fathieh *et al*. [44] reported that Lp(a) >22 nmol/L was significantly associated with multivessel (OR = 1.11) and multisegment disease (OR = 1.14), as well as coronary artery calcium scores >100. Lp(a) also showed the strongest correlation with mixed plaque burden (β = 4.75, *p* = 0.001), rather than with exclusively calcified or non-calcified plaques. Niccoli *et al*. [45] demonstrated that patients with ACS and elevated Lp(a) had a higher prevalence of TCFA and lipid arc burden. Similarly, in a large prospective cohort, Yu *et al*. [46] found that low-attenuation plaques (LAPs), indicative of large lipid cores and thin fibrous caps, were more common in individuals with Lp(a) ≥50 mg/dL and positively correlated with Lp(a) levels (standardized β = 0.35, *p* < 0.001). Elevated Lp(a) was linked to a significantly increased risk of myocardial infarction (MI; 26.1% vs. 6.5%, *p* < 0.001), with LAP mediating approximately 73% of the Lp(a)-MI association. Additional studies by Mszar *et al*. [47] and O’Toole *et al*. [48] confirmed that high Lp(a) is associated with multiple high-risk plaque features—such as TCFA, low attenuation, positive remodeling, and napkin-ring sign—even in subclinical individuals with zero coronary calcium scores.

Current evidence consistently supports a strong link between elevated Lp(a) and coronary plaque vulnerability. Lp(a) is notably associated with key high-risk plaque features detected by OCT (thin caps, lipid arcs), IVUS/NIRS (lipid-rich cores), and CCTA (mixed and low-attenuation plaques). These effects may be mediated by lipid accumulation, oxidative stress, inflammatory responses, and impaired fibrinolysis. While Lp(a)’s impact appears predominantly focal rather than diffuse, further validation is warranted. Future longitudinal studies incorporating multimodal imaging are needed to clarify the role of Lp(a) in plaque evolution and cardiovascular events. With Lp(a)-lowering therapies, such as antisense oligonucleotides and siRNA, advancing in clinical trials, their potential to stabilize high-risk plaques and reduce major adverse cardiovascular events merits close investigation.

Epidemiological studies have established that elevated Lp(a) contributes to the development of ASCVD and CAVD [53]. In patients with advanced stable CHD, high Lp(a) is associated with the rapid progression of low-attenuation plaques (necrotic core) in coronary arteries [49]. Lp(a) levels vary across racial groups, as shown in the INTERHEART study, which found that while elevated Lp(a) is linked to a higher risk of MI, it has a particularly strong effect in Latinos and South Asians, but a weaker association in Arabs and Africans [54]. The BiomarCaRE study, involving seven European cohorts with 56,804 participants followed for up to 24 years, also highlighted regional differences in Lp(a) levels. Elevated Lp(a) is strongly associated with an increased risk of coronary events and overall CVD, particularly in diabetic patients, providing a basis for targeting high-risk populations with Lp(a)-based therapies [55]. Thus, Lp(a) serves as a crucial target for therapeutic intervention in a significant portion of high-risk individuals.

## 6. New Strategies for the Management of High-Risk Coronary Artery Plaques Targeting Lipoprotein(a)

International guidelines have yet to reach a consensus on Lp(a) testing strategies and risk thresholds. The 2023 American College of Cardiology (ACC) and American Heart Association (AHA) guidelines recommend Lp(a) screening for patients with familial ASCVD, considering levels ≥50 mg/dL as an elevated risk factor [56]. The European Society of Cardiology/European Atherosclerosis Society (ESC/EAS) guidelines advocate for a one-time lifetime test, defining Lp(a) >180 mg/dL as high risk. Studies also suggest monitoring adults with marginally elevated Lp(a), particularly Black individuals, women, or those with diabetes, hypertension, or proteinuria [57]. Most perspectives emphasize that Lp(a) measurement can effectively assess ASCVD risk, recommending that most individuals undergo a single Lp(a) test during their lifetime to estimate ASCVD risk [58], particularly those with a family history of early-onset ASCVD. The combination of inflammatory markers for verification is also recommended. The Bruneck study demonstrated that Lp(a) level measurement enhances patient risk prediction and optimizes CVD risk stratification.

High-risk individuals benefit from statin therapy, with the 2025 ACC/AHA guidelines for ACS emphasizing intensified LDL-C management. All patients should start high-intensity statins (atorvastatin 40–80 mg/d), targeting LDL-C <55 mg/dL or a ≥50% reduction. For those not meeting targets, combination therapy with ezetimibe, PCSK9 inhibitors, or bempedoic acid is recommended [59]. While current guidelines do not specify Lp(a)-targeted treatment, the importance of Lp(a) in managing MACE is increasingly recognized.

Lp(a) is a well-established independent risk factor for cardiovascular diseases. Among the lipid-lowering drugs currently in clinical use, only a limited number exhibit the added benefit of reducing Lp(a), which is often regarded as a supplementary effect when lowering LDL-C. While specific therapies targeting Lp(a) are under investigation, the only Food and Drug Administration (FDA)-approved treatment, lipoprotein apheresis (LA), remains accessible only to select patient populations in a few countries.

A pooled study of seven randomized controlled trials (RCTs) (n = 29,069) showed that while statin treatment reduced LDL-C by 39%, Lp(a) levels remained stable. Residual risk was dose-dependently related to Lp(a), confirming its independent predictive value in statin-treated populations, and supporting the development of Lp(a)-lowering therapies [60]. The JUPITER trial (n = 9612) further demonstrated that despite rosuvastatin significantly reducing LDL-C, Lp(a) remained an independent determinant of residual cardiovascular risk, with no racial differences, highlighting the need for targeted interventions to reduce ASCVD risk post-statin treatment [61]. Notably, a meta-analysis (n = 5256) found that high-intensity statins, such as atorvastatin, can increase Lp(a) levels, suggesting that statins may exacerbate Lp(a)-related cardiovascular residual risk via a dose-dependent mechanism [62]. Patients with high Lp(a) levels may need to avoid excessive intensification of statin therapy, highlighting the need for targeted intervention to optimize lipid management strategies.

Studies indicate that elevated Lp(a) levels, independent of LDL-C, significantly increase atherosclerotic risk [63]. Genetic prediction analyses reveal a linear dose-response relationship between Lp(a) and cardiovascular risk: a 10 mg/dL reduction in Lp(a) lowers CHD risk by 5.8% (OR = 0.942), while a 10 mg/dL decrease in LDL-C reduces risk by 14.5% (OR = 0.855). Equivalence analysis suggests that lowering Lp(a) by about 101.5 mg/dL provides the same risk reduction as decreasing LDL-C by 38.67 mg/dL (1 mmol/L). Furthermore, the Lp(a)-CHD risk relationship remains unaffected by statins, PCSK9 inhibitors, and ezetimibe’s impact on LDL-C. Clinically meaningful benefits require a significant reduction in Lp(a) (around 100 mg/dL). Even with effective LDL-C reduction, elevated Lp(a) levels continue to be linked with a higher risk of CVD, likely through mechanisms independent of traditional lipid-lowering pathways [64]. The UK Biobank study (n = 385,917) found that reducing Lp(a) by 50 mg/dL lowers peripheral arterial (PAD) risk (HR = 0.73) and venous thromboembolism risk (HR = 0.95), with no synergistic effect from LDL-C control or lifestyle interventions [65]. These results suggest that Lp(a)-targeted therapies can complement current cardiovascular risk management and support the need for broader Lp(a) testing to identify high-risk patients [66].

Drugs aimed at reducing LDL-C, including PCSK9 inhibitors, show significant potential for lowering Lp(a). In the FOURIER trial (n = 25,096), evolocumab reduced Lp(a) by 26.9% and decreased MACE risk by 23% in patients with high baseline Lp(a) levels, independent of LDL-C [67]. Post hoc analyses from the FOURIER and ODYSSEY OUTCOMES trials consistently demonstrated that PCSK9 monoclonal antibodies (mAbs) significantly reduce plasma Lp(a) levels by approximately 20% to 30%, leading to a reduction in CVD risk independent of LDL-C lowering. Notably, this effect was more pronounced in patient subgroups with higher baseline Lp(a) levels and greater reductions in Lp(a) [68, 69]. Another RCT (NCT03570697) involving NSTEMI patients showed that evolocumab combined with statins significantly reduced LDL-C and improved coronary artery plaque stability, as evidenced by increased minimum fibrous cap thickness, significant plaque volume regression, decreased macrophage index, and reduced maximum lipid arc [70]. A retrospective analysis evaluated the effect of evolocumab on plaque stabilization in patients with varying baseline Lp(a) levels. In those with elevated Lp(a) (≥125 nmol/L), evolocumab significantly reduced LDL-C and Lp(a) levels, and led to greater increases in fibrous cap thickness and reductions in lipid arc compared to placebo. In contrast, among patients with low Lp(a), evolocumab lowered lipids but had no significant impact on plaque composition. A significant interaction was observed between baseline Lp(a) and changes in fibrous cap thickness, suggesting enhanced plaque-stabilizing effects of evolocumab in patients with high Lp(a) [71].

A healthy lifestyle is the primary measure for preventing ASCVD and plays a crucial role in regulating blood lipid [56, 59]. However, Lp(a) concentration is mainly determined by genetic factors and is less influenced by exercise and diet [72]. Weight loss, a relatively high intake of saturated fatty acids, wine consumption, and vigorous exercise appear to correlate with reduced plasma Lp(a) levels. This effect is more pronounced in patients with higher baseline Lp(a) levels. In contrast, regular moderate physical activity does not seem to significantly influence plasma Lp(a) concentrations [73]. Therapeutic lifestyle changes (TLCs) are effective for CVD prevention. Lp(a) levels are negatively correlated with dietary saturated fatty acids (SFA) intake, suggesting SFA affects Lp(a) through epigenetic regulation of lipid metabolism. Healthy lifestyle indicators, such as fish intake, body mass index (BMI), whole grain intake, and reduced sodium and sugar intake, contribute to better Lp(a) management [74]. Regular moderate-intensity exercise improves lipoprotein metabolism but has no significant effect on Lp(a). High-intensity weight-bearing training may increase Lp(a), though its clinical significance remains unclear. Given the synergistic risks of LDL-C and Lp(a), lifestyle adjustments to lower LDL-C and increase HDL-C are recommended. Moderate aerobic exercise and lifestyle changes, such as controlling blood pressure, normalizing blood glucose, quitting smoking, and reducing alcohol intake, enhance cardiovascular health. Although the precise influence of lifestyle on Lp(a) levels is still unclear, an 8-year cohort study revealed during follow-up that a healthy lifestyle was significantly correlated with a lower risk of cardiovascular disease, irrespective of Lp(a) concentrations [75].

Given the limited efficacy of conventional therapies in managing Lp(a)-related residual risk, the need for targeted Lp(a)-lowering strategies has become increasingly urgent. Recently, several RNA-based therapeutics (e.g., Pelacarsen, Olpasiran, SLN360) have entered clinical trials, showing marked reductions in Lp(a) levels and preliminary evidence of safety and benefit.

Antisense oligonucleotides (ASOs) are short, single-stranded DNA sequences that can hybridize with target mRNA to form ASO-RNA duplexes. Through the mechanism of steric hindrance and ribonuclease H-mediated degradation of the RNA strand, ASOs modulate the expression of specific molecules. Early-generation ASO drugs targeting Lp(a) clearance included ISIS-APO(a)Rx and IONIS-APO(a)Rx. Pelacarsen, as a second-generation IONIS-APO(a)Rx drug, exhibits enhanced durability and stability [76]. Results from a Phase IIb trial demonstrated that monthly subcutaneous injections of 80 mg over 6 months reduced median Lp(a) levels by 80%, with 98% of patients achieving Lp(a) levels below 50 mg/dL and showing favorable safety profiles. The global Phase III trial (NCT04023552), enrolling 8300 patients with cardiovascular disease and Lp(a) ≥70 mg/dL, aims to evaluate the long-term effects of Lp(a) reduction on cardiovascular outcomes and is expected to conclude in 2025. Studies confirm that Pelacarsen significantly lowers direct Lp(a)-cholesterol, with corrected LDL-C providing a more accurate reflection of lipid changes [77]. Furthermore, the HORIZON trial represents the first systematic investigation into the dose-response relationship between Lp(a) reduction and cardiovascular benefits.

Olpasiran, a GalNAc-conjugated siRNA molecule, inhibits Lp(a) particle assembly by degrading apo(a) mRNA and blocking *LPA* gene expression. Phase I trials showed dose-dependent reductions in Lp(a) of >90% at doses ≥9 mg, with effects lasting 3–6 months. In the phase II OCEAN(a)-DOSE study (n = 281, ASCVD patients with Lp(a) ≥150 nmol/L), Olpasiran led to placebo-adjusted reductions of 70.5% to 101.1%, with the greatest reduction in the 225 mg/12 weeks group. It was well tolerated, with mild injection site pain as the primary adverse effect [78]. Extended follow-up showed sustained Lp(a) reductions of 40%–50% up to one year post-treatment in patients receiving ≥75 mg every 12 weeks [78]. In June 2023, Olpasiran received breakthrough therapy designation from the Center for Drug Evaluation (CDE) for reducing CHD death, MI, and emergency revascularization risk in ASCVD patients. Phase III clinical trials of Olpasiran (NCT05581303) have commenced, aiming to enroll 7000 ASCVD patients with elevated Lp(a) levels. The trial results are expected to be released in December 2026.

Lepodisiran is a long-acting GalNAc-conjugated siRNA that degrades apo(a) mRNA via the RNA-induced silencing complex (RISC) after entering liver cell nuclei, thereby inhibiting Lp(a) synthesis. A first-in-human study showed that a single 608 mg dose reduced Lp(a) by 97% at 48 weeks, with reductions maintained at 40%–50% through week 48. There were no serious adverse events during the study period, and the drug was well-tolerated. A phase III trial is underway in China to evaluate its efficacy in reducing MACE in ASCVD patients with elevated Lp(a) [79].

SLN360, a highly selective siRNA, significantly reduced Lp(a) by 96% after a single 600 mg injection, with effects lasting over 6 months. The drug was well tolerated, with no serious adverse events reported. It demonstrated potent and durable inhibition of Lp(a), leading to a phase III trial for high-risk ASCVD patients [80].

Muvalaplin (LY3473329) is the first oral drug targeting Lp(a) assembly. It inhibits Lp(a) formation by disrupting the interaction between apo(a) and apoB. Phase II trials showed that daily 30–100 mg doses for 12 weeks reduced Lp(a) by 65%, significantly lowering levels in high-risk cardiovascular patients [81].

Additionally, Lipoprotein apheresis (LA) has become an important therapeutic option for patients with drug-resistant angina and elevated Lp(a), providing benefits such as improved myocardial perfusion, reduced atherosclerotic burden, and enhanced exercise capacity [82]. The use of LA varies across countries; in Spain, the UK, and Japan, it is restricted to patients with homozygous or heterozygous familial hypercholesterolemia (HoFH/HeFH), whereas in Germany and the US, its indications have been extended to include drug-resistant Lp(a)-elevated CVD [83, 84]. Despite these advancements, LA’s clinical application remains limited by its invasiveness (requiring 1–2 sessions per week, lasting 2–4 hours each), high cost ($2000 per session), and potential adverse effects (including hypotension and allergic reactions in approximately 15% of cases). The ongoing MultiSELECT Phase III trial (NCT02791802) aims to evaluate the efficacy of LA in reducing MACEs compared to maximum-tolerated lipid-lowering therapy. Current evidence indicates that until emerging Lp(a)-targeted therapies like Pelacarsen and Olpasiran become widely available, LA remains a key intervention for rapidly lowering Lp(a) levels (with reductions of 60%–75% per session).

Clinical studies have shown that LDL-C levels are inversely associated with mortality risk in AMI patients, especially those with high inflammatory risk, a phenomenon known as the “lipid paradox”. Statins not only lower LDL-C but also exert anti-inflammatory effects, which may mitigate the lipid paradox. These findings emphasize the need for personalized lipid-lowering strategies based on inflammatory status [85].

The OCEAN(a)-DOSE trial (n = 53) showed significant variability in Lp(a) measurements (CVi = 10%) among ASCVD patients with Lp(a) >150 nmol/L. This suggests that multiple measurements are necessary, particularly near critical thresholds, to guide treatment effectively [86].

The structural diversity of Lp(a) influences its particle size, which in turn affects measurement outcomes. Variability in the number of KIV-2 repeat sequences within apo(a) leads to substantial differences in particle size [87]. Furthermore, due to molecular weight differences among apo(a) isoforms, there is no fixed conversion factor between mass-based units (mg/dL) and particle-based units (nmol/L) [88]. Traditional immunoassays may underestimate small-particle isoforms and overestimate large-particle isoforms due to cross-reactivity or misrecognition of repetitive structural motifs. Additionally, discrepancies in calibrator isoform composition across commercial assay kits contribute to poor inter-assay comparability, particularly in populations with elevated Lp(a) levels [89].

Current strategies and guidelines addressing these challenges include the use of isoform-insensitive assays that target non-KIV-2 regions of apo(a), such as the KV domain, to ensure each Lp(a) molecule is detected only once. The WHO/International Federation of Clinical Chemistry and Laboratory Medicine (IFCC) recommends the use of assays calibrated with the WHO/IFCC reference material (SRM 2B), including ELISA or turbidimetric methods [90]. However, fully isoform-independent commercial assays are not yet available, and users should verify whether the manufacturer specifies “isoform insensitivity” in the product documentation. Unit selection is also critical: in clinical settings, particle concentration expressed in nmol/L is preferred over mass concentration in mg/dL, as cardiovascular risk correlates more closely with Lp(a) particle number. Due to the structural heterogeneity of Lp(a), thresholds derived from different studies (e.g., 50 mg/dL vs. 125 nmol/L) cannot be directly interchanged and must be interpreted according to their original measurement units.

The management strategy for HRPs targeting Lp(a) primarily includes: ① Lipid management: combined use of statins and ezetimibe, with PCSK9 inhibitors if necessary. However, statins may increase plasma Lp(a) levels, warranting investigation into their impact on residual cardiovascular risk. ② Family screening: first-degree relatives should undergo Lp(a) testing. ③ Anti-thrombotic therapy: aspirin may be considered for high-risk individuals [62]. ④ Imaging monitoring: regular assessment of atherosclerotic plaque progression optimizes Lp(a)-targeted high-risk coronary plaque management strategies. Recent advances in Lp(a)-lowering therapies, including antisense oligonucleotides and siRNA-based approaches, offer promising potential for reducing residual cardiovascular risk (Table 2).

**Table 2.** **Clinical research progress of Lp(a) -related drugs**.

Drug	Mechanism	Route	Key Trial	Peak efficacy	Frequency	Safety profile	Phase III status
Pelacarsen	Second-gen ASO (IONIS-APO(a)Rx)	Subcutaneous	Phase IIb + HOR IZON	80% (median)	Monthly	Favorable, no SAEs	NCT04023552, completion 2025
Olpasiran	GalNAc-conjugated siRNA	Subcutaneous	OCEAN(a)-DOSE (Phase II)	>90% (highest dose)	Every 12 weeks	Mild injection site pain	NCT05581303, completion Dec 2026
Lepodisiran	Long-acting GalNAc-siRNA	Subcutaneous	First-in-human	97% at 48 weeks	Single dose for 48 weeks	Well tolerated	Phase III ongoing (China)
SLN360	Highly selective siRNA	Subcutaneous	First-in-human	96% (single 600 mg dose)	>6 months	No SAEs reported	Phase III initiated (high-risk ASCVD)
Muvalaplin	Oral small molecule (apo(a)-apoB disruptor)	Oral	Phase II	65% (12 weeks)	Daily	Well tolerated	Phase III has not yet been initiated

ASO, Antisense oligonucleotide.

## 7. Perspectives

Future research should focus on elucidating the functional mechanisms of Lp(a) in coronary HRPs, including its associations with plaque instability, hemodynamic changes, and intercellular interactions. Simultaneously, by integrating high-resolution imaging technologies and multi-omics analyses, the correlations between Lp(a) levels and plaque structural and functional indicators should be explored to refine measurement methodologies and enhance risk assessment models. The efficacy of combining Lp(a)-targeted therapies (e.g., ASOs, RNA interference) with conventional treatments (lipid-lowering, anti-inflammatory, and antithrombotic therapies) should be evaluated. Additionally, further investigation is needed on the impact of statin-induced Lp(a) elevation on residual cardiovascular risk. It is essential to integrate existing treatment modalities to assess their long-term clinical benefits and safety, thereby providing robust evidence-based support for reducing the risk of coronary events.

In summary, Lp(a) holds great potential as a key biomarker and therapeutic target for coronary HRPs. Advances in omics, big data, and personalized medicine are expected to improve risk assessment, treatment strategies, and outcomes, reducing acute coronary event incidence.

## 8. Conclusion 

In summary, converging evidence from genetic, mechanistic, and intravascular imaging studies has firmly established lipoprotein(a) as both a biomarker and a causal mediator of coronary plaque vulnerability. Elevated Lp(a) promotes lipid accumulation, inflammatory activation, and prothrombotic changes within the arterial wall, leading to accelerated plaque progression and destabilization. Advanced imaging modalities, including OCT, IVUS, and CCTA, have provided *in vivo* validation that high Lp(a) levels are strongly associated with thin-cap fibroatheromas, enlarged lipid cores, and features of neoatherosclerosis—linking molecular abnormalities to structural plaque instability.

Despite contemporary lipid-lowering therapies, the residual cardiovascular risk attributable to elevated Lp(a) remains substantial. These insights highlight the necessity of integrating molecular characterization with plaque imaging to enable precise risk stratification and tailored therapeutic interventions. Such integration will be crucial for transforming our understanding of Lp(a)-driven atherogenesis into clinically actionable strategies.
